# Controlling stick balancing on a linear track: Delayed state feedback or delay-compensating predictor feedback?

**DOI:** 10.1007/s00422-023-00957-w

**Published:** 2023-03-21

**Authors:** Dalma J. Nagy, John G. Milton, Tamas Insperger

**Affiliations:** 1 Department of Applied Mechanics, Faculty of Mechanical Engineering, Budapest University of Technology and Economics, Budapest, Hungary; 2 W. M. Keck Science Center, Claremont Colleges, Claremont, CA 91711 USA; 3 ELKH-BME Dynamics of Machines Research Group, Budapest, Hungary

**Keywords:** Human balancing, Reaction delay, Delayed feedback, Stabilometry, Certainty threshold

## Abstract

A planar stick balancing task was investigated using stabilometry parameters (SP); a concept initially developed to assess the stability of human postural sway. Two subject groups were investigated: 6 subjects (MD) with many days of balancing a 90 cm stick on a linear track and 25 subjects (OD) with only one day of balancing experience. The underlying mechanical model is a pendulum-cart system. Two control force models were investigated by means of numerical simulations: (1) delayed state feedback (DSF); and (2) delay-compensating predictor feedback (PF). Both models require an internal model and are subject to certainty thresholds with delayed switching. Measured and simulated time histories were compared quantitatively using a cost function in terms of some essential SPs for all subjects. Minimization of the cost function showed that the control strategy of both OD and MD subjects can better be described by DSF. The control mechanism for the MD subjects was superior in two aspects: (1) they devoted less energy to controlling the cart’s position; and (2) their perception threshold for the stick’s angular velocity was found to be smaller. Findings support the concept that when sufficient sensory information is readily available, a delay-compensating PF strategy is not necessary.

## Introduction

Neuroscientists often take for granted the hypothesis that the nervous system uses an internal model to predict the sensory consequences of a movement (Jordan 1996; Huang et al. 2018) Internal models can either be used to generate a desired motion by feedforward motor commands based on inverse dynamics in an open-loop mechanism (often referred to as feedforward model) (Jordan 1996; Kawato 1999; Wolpert and Ghahramani 2000) or to design a feedback loop for error correction (Wolpert et al. 1995; Jordan 1996; Mehta and Schaal 2002). In the latter case, internal model is used either to design control gains for optimal error-correcting feedback control, or to predict the outcome of an action before sensory feedback is available by integrating motor efference copies in order to reduce the effect of feedback delays (Wolpert et al. 1995; Jordan 1996; Todorov and Jordan 2002; Mehta and Schaal 2002).

Stick balancing is a voluntary motor skill in which theoretical predictions for the stabilization of an inverted pendulum can be evaluated experimentally (Reeves et al. 2013; Gawthrop et al. 2013; Yoshikawa et al. 2016). Longer sticks are easier to balance than shorter ones. This observation emphasizes that a time delay, $\tau$, plays a major role in the feedback control (Milton et al. 2016; Insperger and Milton 2021). The presence of a time delay means that sensory information obtained in the past is used to generate corrective actions made in the present.

The important question is to determine the nature of the feedback control action. Delayed state feedback (DSF) controllers feed back information about the state as determined by its position, velocity and acceleration measured at time $t-\tau$. On the other hand, internal-model-based predictor feedback (PF) controllers predict the actual state of the system at time *t* based on the known delayed state information at time $t-\tau$ and the known control command history over the interval between $t-\tau$ and *t* (Insperger and Milton 2014, 2021; Krstic 2009).

What are the factors that determine whether the nervous system uses a PF strategy or DSF to control a motor task? A simple hypothesis is that the nervous system uses PF to control a motor task whenever the relevant sensory information cannot adequately be measured. Here we examine the hypothesis in the context of human stick balancing (Insperger and Milton 2021). We demonstrate that PF is not necessary for balancing tasks when sufficient sensory information is available to the nervous system. In such cases, DSF with delayed feedback motor commands is sufficient.

A previous study demonstrated that for stick balancing at the fingertip, expert stick balancers use PF (Milton et al. 2016). For stick balancing at the fingertip there is a large sensory dead zone in the anterior-posterior (AP) direction that primarily arises because of limitations in depth perception. Here we minimize the effects of depth perception by identifying the feedback for stick balancing on a linear track. For linear track stick balancing, the movements are confined to the medio-lateral (ML) direction. A consequence is that sensory dead zones are much smaller. This balancing task has been extensively studied previously in order to determine the nature of the perceptual information used by the nervous system and to examine coordination dynamics (de Guzman 2004; Treffner and Kelso 1999; Foo et al. 2000). However, to our knowledge the nature of the feedback has not yet been identified for this task.

**Fig. 1:**
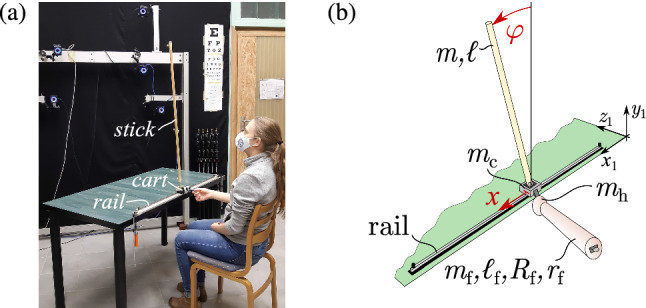
**a** Stick balancing on a linear track. The stick is pinned to the cart, which is constrained to move along the rail. Subjects were asked to sit on a chair so that their shoulders were parallel to the rail, and balance the stick in the ML direction with their dominant hand using the handle of the cart. **b** Two degree of freedom mechanical model of the stick balancing task, where the generalized coordinates are $\varphi$ and *x*. The stick exerts planar motion in the $(x_1,y_1)$ plane. The mass and length of the stick are *m* and $\ell$, respectively. The mass and height of the truncated cone for modeling the human forearm are $m_{\textrm{f}}$ and $\ell _{\textrm{f}}$, the radius of the base and top circles are $r_{\textrm{f}}$ and $R_{\textrm{f}}$, respectively. The mass of the hand and of the cart are $m_{\textrm{h}}$ and $m_{\textrm{c}}$

In this paper, a mechanical model for stick balancing on a linear track shown in Fig. 1 is developed. The state of the stick as well as the cart are fed back into the control loop. Feeding back the state of the cart alters the stability properties of the system compared to the case when only the state of the stick is considered in the feedback loop. Thresholds and reaction delay are taken into account when developing the mechanical models leading to the application of hybrid control systems with switching and time delay. Two general types of feedback for stabilizing the stick are considered: (1) * delayed state feedback (DSF)*, namely feedback that depends on the displacement angle and its rate of change (Stepan 1989), and (2) *predictor feedback (PF)*, which includes a perfect internal model that incorporates a time delay (Krstic 2009). We determine the roles played by DSF and PF in stick balancing. The feedback models are validated by systematic series of stick balancing trials involving 31 subjects. The models and the actual balancing trials are compared by means of stabilometry; an objective tool initially introduced to study body sway during quiet standing and during different balancing exercises (Petró et al. 2017; Nagymáté et al. 2018; Molnar and Insperger 2020; Molnar et al. 2021).

Our results demonstrate that humans use DSF to control stick balancing in this task. Taken together the feedback identifications for stick balancing at the fingertip and on a linear track suggest that a PF strategy may be required in situations in which a significant amount of sensory information is unavailable.

The outline of the paper is as follows. Section 2 explains the feedback models applied and their stability analysis. Section 3 describes the numerical and measurement methods together with the stabilometry-based data analysis. Section 4 compares the time histories given by numerical simulation corresponding to the feedback models to the time histories recorded during the balancing trials. Section 5 summarizes the findings of the study regarding the feedback models, time delay and threshold values.

## Stick balancing on a horizontal track

Figure 1 shows stick balancing on a horizontal track used in our investigations. This setup is very similar to that used in previous studies (de Guzman 2004; Foo et al. 2000; Reeves et al. 2013; Treffner and Kelso 1999). The subject controls the vertical displacement angle of the stick, $\varphi$, by using a handle to control the movements of a cart confined to move along a linear track (Fig. 1a). An equivalent mechanical model is shown in Fig. 1b. The model assumes that the displacements of the elbow during balancing are negligible.

The equation of motion was derived using the generalized coordinates *x* and $\varphi$. The inertia of the cart, hand and forearm is replaced by one single cart with an equivalent mass $m_{\textrm{e}}$, which can be determined using the equivalence of kinetic energy similarly to Nagy et al. (2020). This determination assumes that the hand can be modeled as a point mass and the forearm as a truncated cone (Hanavan 1964). Using anthropometric data (de Leva 1996), we obtain $m_{\textrm{e}}=1.73$ kg. The linearized equation of motion around $x=0$ and $\varphi =0$ is

1 $$\begin{aligned} \begin{bmatrix} m+m_{\textrm{e}} &{}\quad \frac{m \ell }{2} \\ \frac{m \ell }{2} &{}\quad \frac{m \ell ^2}{3} \end{bmatrix} \! \begin{bmatrix} \ddot{x} \\ \ddot{\varphi }\end{bmatrix} \!+\! \begin{bmatrix} 0 &{}\quad 0 \\ 0 &{}\quad -\frac{mg \ell }{2} \end{bmatrix} \! \begin{bmatrix} x \\ \varphi \end{bmatrix} \!=\! \begin{bmatrix} F(t) \\ 0 \end{bmatrix}, \end{aligned}$$

where *F*(*t*) is the control force exerted by the human subject and *g* is the gravitational acceleration. In this study, the length and the mass of the stick were $\ell =90$ cm and $m=0.10$ kg.

If the control force *F*(*t*) is zero, then the state variables can be separated and the motion of the stick is governed purely by

2 $$\begin{aligned} \ddot{\varphi }- \frac{6g}{c\ell } \varphi =0, \end{aligned}$$

where $c=4-3m/(m+m_{\textrm{e}})$ with $1 \le c \le 4$. In this case, the cart position becomes a cyclic coordinate, which can be determined from

3 $$\begin{aligned} \ddot{x}= -\frac{m\ell }{2(m+m_{\textrm{e}})} \ddot{\varphi }. \end{aligned}$$

It can be seen that the solution $\varphi =0$ of Eq. (2) is unstable due to the negative gravitational stiffness term $-6g/(c\ell )$. On the other hand, the position of the cart is governed by Eq. (3), which is a marginally stable system (double integrator) due to the lack of any stiffness or damping term. In this sense, the primary (critical) task of the stabilization problem is keeping the stick in the $\varphi =0$ position and the positioning of the cart to $x=0$ is a secondary (“not so critical”) task.

In order to keep the stick in the vertical position, active feedback control is required. It is assumed that the control action is determined by the linear combination of the state variables $\varphi ,{\dot{\varphi }}, x, \dot{x}$, which is referred as proportional-derivative (PD) feedback (Mauer and Peterka 2005; Stepan 2009; Gawthrop et al. 2013; Yoshikawa et al. 2016; Insperger and Milton 2021). The control force is written as

4 $$\begin{aligned} F(t) = F_{\varphi }(t) + F_{\dot{\varphi }}(t) + F_x(t) + F_{\dot{x}}(t), \end{aligned}$$

where $F_{\varphi }(t), F_{\dot{\varphi }}(t),F_x(t), F_{\dot{x}}(t)$, respectively correspond to the feedback terms originated from $\varphi ,{\dot{\varphi }}, x, \dot{x}$.

A key feature of human balancing is the delay in the feedback due to the fact that for the nervous system it takes finite time to process sensory information and initiate action. However, there is evidence that humans are capable of making a prediction by integrating motor efference copies using an internal model in order to compensate this delay (Wolpert et al. 1995; Kawato 1999; Desmurget and Grafton 2000; Mehta and Schaal 2002; Milton et al. 2016; Insperger and Milton 2021). This suggests two different control concepts:

1. Delayed state feedback (DSF);
2. Predictor feedback (PF).

In case of DSF, the delayed state variables are directly fed back and the control force reads

5 $$\begin{aligned} F_{\textrm{DSF}}(t)= & {} P_{\textrm{s}} \varphi (t-\tau )+D_{\textrm{s}} {\dot{\varphi }}(t-\tau ) \nonumber \\{} & {} + P_{\textrm{c}} x(t-\tau )+D_{\textrm{c}} \dot{x}(t-\tau ), \end{aligned}$$

where $\tau$ is the reaction delay. The parameters $P_{\textrm{s}}$ [N/rad] and $D_{\textrm{s}}$ [Ns/rad] stand for the proportional and derivative gains with respect to the stick, while $P_{\textrm{c}}$ [N/m] and $D_{\textrm{c}}$ [Ns/m] stand for the proportional and derivative gains with respect to the cart.

In case of PF the reaction delay is compensated by predicting the state over the delay period based on an internal model. The corresponding control force reads

6 $$\begin{aligned} F_{\textrm{PF}}(t)= & {} P_{\textrm{s}} \varphi (t-\tau )+D_{\textrm{s}} {\dot{\varphi }}(t-\tau ) + P_{\textrm{c}} x(t-\tau ) \nonumber \\{} & {} +D_{\textrm{c}} \dot{x}(t-\tau ) + \int _{t-\tau }^{t} k_{\textrm{f}}(t-s) F_{\textrm{PF}}(s) \textrm{d}s, \end{aligned}$$

where the integral term is associated with the information provided by the efferent copies and $k_{\textrm{f}}$ is an exponential function obtained by solving the internal model equation (Insperger and Milton 2021). If the integral term in (6) is zero then we get (5). Therefore, DSF can also be considered as a predictor feedback with an imperfect internal model, where the present state is approximated directly by the delayed state. In case of a perfect internal model and a perfect implementation of (6), the delay can fully be compensated and the PF control force can be written as

7 $$\begin{aligned} F_{\textrm{PF}}(t) = {\hat{P}}_{\textrm{s}} \varphi (t)+{\hat{D}}_{\textrm{s}} {\dot{\varphi }}(t) + {\hat{P}}_{\textrm{c}} x(t)+{\hat{D}}_{\textrm{c}} \dot{x}(t), \end{aligned}$$

where ${\hat{P}}_{\textrm{s}}, {\hat{D}}_{\textrm{s}}, {\hat{P}}_{\textrm{c}}, {\hat{D}}_{\textrm{c}}$ are linear combinations of $P_{\textrm{s}}$, $D_{\textrm{s}}$, $P_{\textrm{c}}$, $D_{\textrm{c}}$ and depend also on the internal model (Insperger and Milton 2021). Note that perfect prediction is not possible due to sensory uncertainties, imperfections of the internal model and motor noise. In this paper, these uncertainties will be modeled by an actuation dead zone (see Sect. 2.2).

**Fig. 2:**
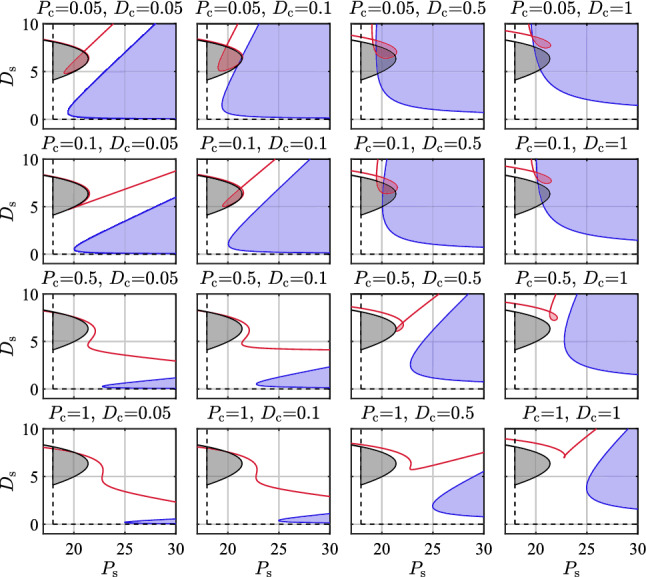
Linear stability diagrams for a stick of length $\ell =90$ cm controlled by DSF and by PF for feedback delay $\tau =230$ ms. The dashed black curves show the stability boundaries for PF while $P_{\textrm{c}}=D_{\textrm{c}}=0$, the stable domain in this case is the quarter plane defined by $P_{\textrm{s}}\ge g(m+m_{\textrm{e}})$ and $D_{\textrm{s}}\ge 0$. The solid black curves show the stability boundaries for DSF while $P_{\textrm{c}}=D_{\textrm{c}}=0$ and the stable region is shown by gray shading. The blue and the red curves show the D-curves for PF and DSF respectively, while $P_{\textrm{c}}$ and $D_{\textrm{c}}$ are set according to the label above each panel. Reddish and blueish shaded areas indicate the corresponding stable domains

Figure 2 shows a series of stability diagrams for (1) either with (5) or with (7) obtained by the method of D-curves (Stepan 1989). The size of the stable regions reflects the robustness to parameter uncertainties. It can be seen that the stable regions get smaller with increasing $P_{\textrm{c}}$ and $D_{\textrm{c}}$ for both DSF and PF. Hence, the more control effort is devoted to controlling the cart, the smaller the chance to control the stick’s position robustly. Furthermore, it can be observed that the stable region for PF is significantly larger than that for DSF independently whether the control of the cart is active or not. Thus, we anticipate that (perfect) PF provides more robust control when sensory uncertainties are large.

The location of the stable region is also an important feature of the control system from energy efficiency point of view since large control gains result in large control effort. It can also be observed in Fig. 2 that PF allows smaller derivative gain $D_{\textrm{s}}$ than DSF does and the lower limit for the proportional gain $P_{\textrm{s}}$ is about the same. Note that if $P_{\textrm{s}} < g(m+m_{\textrm{e}})=17,95$ N/rad then the system cannot be stabilized for any $(P_{\textrm{c}},D_{\textrm{c}},D_{\textrm{s}})$ triplets for both PF and DSF. Therefore the horizontal axis is plotted in the range $15<P_{\textrm{s}}<30$.

Stability diagrams in Fig. 2 for DSF very much resemble those of Yoshikawa et al. (2016), where the stability diagrams were determined by brute force simulations for the same pendulum-cart model. The goal in Yoshikawa et al. (2016) was to show that a smart state-space-based on–off switching of DSF, referred to as intermittent control, can be used to increase the size of the stable regions by exploiting the stable manifold of the uncontrolled stick.

The exerted control force is affected by sensory and motor uncertainties. For stick balancing on the fingertip the sensory dead zone in the AP direction ranges from $\approx$ 0.75–3.5 degrees (for 8 subjects) and is thought to be largely due to the effects of depth perception (Milton et al. 2016). The deleterious effects of depth perception can be minimized by confining the control movements to a linear track. However, other sources of uncertainty such as those due to the finite resolution of the visual system ($\approx 0.02^{\circ }$ DeValois and DeValois 1990) are still present (see also Discussion). Moreover, it has been suggested that a small dead zone has the beneficial effects of preventing the effects of over-control (Milton et al. 2008).

It is assumed that when the displacement or velocity is smaller than a threshold the nervous system does not generate a response (Milton et al. 2016). Therefore the corresponding control model involves a dead zone.

### Delayed state feedback with dead zone

If dead zones are accounted for in the model, then the terms related to $\varphi$ and $\dot{\varphi }$ in Eq. (4) for the DSF model can be formulated as:

8 $$\begin{aligned}{} & {} F_{\varphi }(t) ={\left\{ \begin{array}{ll} 0, &{} \textrm{if}\quad |\varphi (t-\tau )| < \varPi _{\varphi }, \\ P_{\textrm{s}} \varphi (t-\tau ), &{} \textrm{if}\quad |\varphi (t-\tau )| \ge \varPi _{\varphi }, \end{array}\right. } \end{aligned}$$

9 $$\begin{aligned}{} & {} F_{{\dot{\varphi }}}(t) = {\left\{ \begin{array}{ll} 0, &{} \textrm{if}\quad |{\dot{\varphi }}(t-\tau )| < \varPi _{{\dot{\varphi }}}, \\ D_{\textrm{s}} {\dot{\varphi }}(t-\tau ), &{} \textrm{if}\quad |{\dot{\varphi }}(t-\tau )| \ge \varPi _{{\dot{\varphi }}}, \end{array}\right. } \end{aligned}$$

where $\varPi _{\varphi }$ and $\varPi _{{\dot{\varphi }}}$ are the thresholds for $\varphi$ and $\dot{\varphi }$.

The control terms related to *x* and $\dot{x}$ are assumed to be affected by another effect. Since the control of the cart position can be considered as a secondary control task, it is assumed that the corresponding control action becomes active only if *x* exceeds some limit, i.e., if $|x| \ge \varPi _{x}$. Hence,

10 $$\begin{aligned}{} & {} F_{x}(t) = {\left\{ \begin{array}{ll} 0, &{} \textrm{if}\quad |x(t-\tau )| < \varPi _{x}, \\ P_{\textrm{c}} x(t-\tau ), &{} \textrm{if}\quad |x(t-\tau )| \ge \varPi _{x}, \end{array}\right. } \end{aligned}$$

11 $$\begin{aligned}{} & {} F_{\dot{x}}(t) = {\left\{ \begin{array}{ll} 0, &{} \textrm{if}\quad |x(t-\tau )| < \varPi _{x}, \\ D_{\textrm{c}} \dot{x}(t-\tau ), &{} \textrm{if}\quad |x(t-\tau )| \ge \varPi _{x}, \end{array}\right. } \end{aligned}$$

The interval $(-\varPi _{x},\varPi _{x})$ can be considered as a “convenient zone” where no control action is taken with respect to the cart movement. If the cart leaves the convenient zone, then the control of the cart becomes active. This reflects the instruction that the main control goal is to keep the stick balanced while the cart should not be positioned precisely to the middle of the track. The convenient zone corresponds to the concept of barrier-function-based safety control (Ames et al. 2017; Molnar et al. 2022) in the sense that control actions are initiated in order to prevent the cart from reaching the end of the track. The stability properties of the system with (8)–(11) are investigated via numerical analysis (see Sect. 3).

### Predictor feedback with dead zone

If the control force is based on perfect prediction, then the delay is completely eliminated from the feedback loop. However, if the state variables $\varphi$, ${\dot{\varphi }}$ are less than the corresponding thresholds, then no prediction can be made due to the lack of information about the state in the time interval $(t_{\textrm{in}},\ t_{\textrm{out}})$. Here, $t_{\textrm{in}}$ is the time instant when a state variable enters the dead zone and $t_{\textrm{out}}$ is the time instant when it leaves the dead zone. After leaving the dead zone, detection of the state takes finite time and corrective movements can be made only at time $t_{\textrm{out}}+\tau$, where $\tau$ is the same human reaction delay as in DSF. Thus, the reaction delay is present in the threshold crossing condition. The corresponding control force terms in (4) can be formulated as

12 $$\begin{aligned}{} & {} F_{\varphi }(t) ={\left\{ \begin{array}{ll} 0, &{} \textrm{if}\quad |\varphi (t-\tau )| < \varPi _{\varphi }, \\ {\hat{P}}_{\textrm{s}} \varphi (t), &{} \textrm{if}\quad |\varphi (t-\tau )| \ge \varPi _{\varphi }, \end{array}\right. } \end{aligned}$$

13 $$\begin{aligned}{} & {} F_{{\dot{\varphi }}}(t) = {\left\{ \begin{array}{ll} 0, &{} \textrm{if}\quad |{\dot{\varphi }}(t-\tau )| < \varPi _{{\dot{\varphi }}}, \\ {\hat{D}}_{\textrm{s}} {\dot{\varphi }}(t), &{} \textrm{if}\quad |{\dot{\varphi }}(t-\tau )| \ge \varPi _{{\dot{\varphi }}}, \end{array}\right. } \end{aligned}$$

14 $$\begin{aligned}{} & {} F_{x}(t) = {\left\{ \begin{array}{ll} 0, &{} \textrm{if}\quad |x(t-\tau )| < \varPi _{x}, \\ {\hat{P}}_{\textrm{c}} x(t), &{} \textrm{if}\quad |x(t-\tau )| \ge \varPi _{x}, \end{array}\right. } \end{aligned}$$

15 $$\begin{aligned}{} & {} F_{\dot{x}}(t) = {\left\{ \begin{array}{ll} 0, &{} \textrm{if}\quad |x(t-\tau )| < \varPi _{x}, \\ {\hat{D}}_{\textrm{c}} \dot{x}(t), &{} \textrm{if}\quad |x(t-\tau )| \ge \varPi _{x}. \end{array}\right. } \end{aligned}$$

Note that the delay $\tau$ shows up only in the switching conditions but not in the feedback terms. Similarly to DSF, stability properties of the system with Eqs. (12)–(15) can be investigated numerically (see Sect. 3).

## Methods

Stability analysis of the two feedback models with dead zones were investigated via a numerical brute force method. Measurements with stick balancing subjects were carried out in order to investigate the human performance during balancing.

### Numerical analysis of the models

Numerical analysis was carried out using the semi-discretization method (Insperger and Stepan 2011) on the feedback models given by $F_{\textrm{DSF}}$ and $F_{\textrm{PF}}$ augmented with thresholds. The stick length was set to $\ell =90$ cm and simulations were carried out for a wide range of parameter sets ($P_{\textrm{c}},\ D_{\textrm{c}},\ \varPi _{{\dot{\varphi }}}$, $\varPi _{\varphi }$, $\tau$) according to Table 1. The control gains $P_{\textrm{s}},\ D_{\textrm{s}}$ for the stick were investigated in the range $g(m+m_{\textrm{e}}) \le P_{\textrm{s}} \le 10g(m+m_{\textrm{e}})$ and $0\le D_{\textrm{s}} \le 2(m+m_{\textrm{e}})\sqrt{6cg \ell }$.

**Table 1:** The range of the model parameters applied in the numerical simulations

Parameter	Values
$P_{\textrm{s}}$, ${\hat{P}}_{\textrm{s}}$ [N/rad]	18, 18.5, 19, $\ldots$, 180
$D_{\textrm{s}}$, ${\hat{D}}_{\textrm{s}}$ [Ns/rad]	1, 1.2, 1.4, $\ldots$, 36
$P_{\textrm{c}}$, ${\hat{P}}_{\textrm{c}}$ [N/m]	0.05, 0.1, 0.5, 1
$D_{\textrm{c}}$, ${\hat{D}}_{\textrm{c}}$ [Ns/m]	0.05, 0.1, 0.5, 1
$\varPi _{{\dot{\varphi }}}$ [$^{\circ }/\textrm{s}$]	0, 0.005, 0.01, 0.015, 0.05
	0.1, 0.15, 0.5
$\varPi _{\varphi }$ [$^{\circ }$]	0, 0.005, 0.01, 0.05, 0.1
$\tau$ [ms]	100, 140, 170, 200, 230, 260
	290, 320, 360

The threshold for the cart position was considered to be $\varPi _{x}=0.15$ m, which corresponds to a 30 cm “convenient zone” in the middle of the track: in average 83% of the balancing time the cart was within this zone during the measurements.

**Fig. 3:**
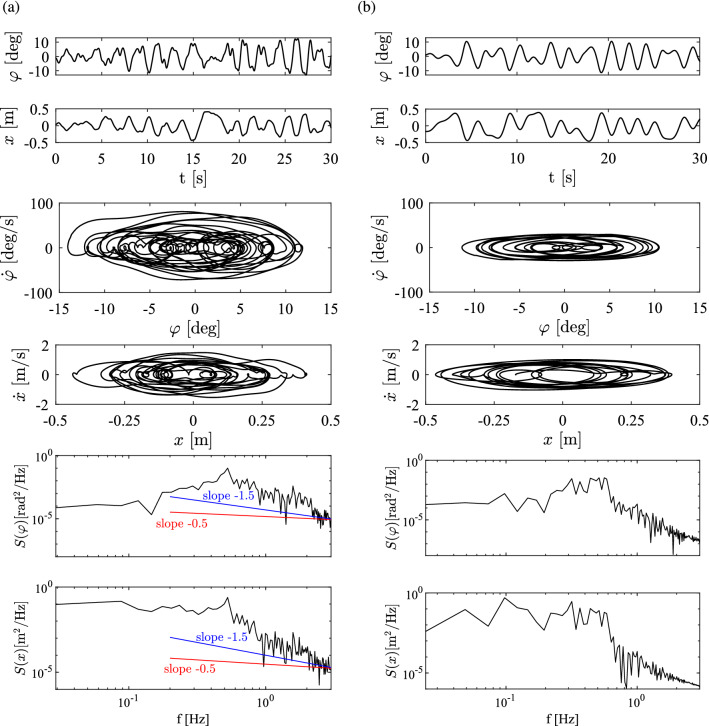
**a** Time histories, phase portraits and PSDs of $\varphi$ and *x* for a representative trial by S21 (OD subject). **b** Time histories, phase portraits and PSDs of the solution provided by the DSF model with parameters identified for S21. Parameters: $P_{\textrm{s}}=22, D_{\textrm{s}}=6.3, P_{\textrm{c}}=0.5, D_{\textrm{c}}=1, \varPi _{{\dot{\varphi }}}=0.05^{\circ }$/s, $\varPi _{\varphi }=0.05^{\circ }$, $\tau =200$ ms

**Fig. 4:**
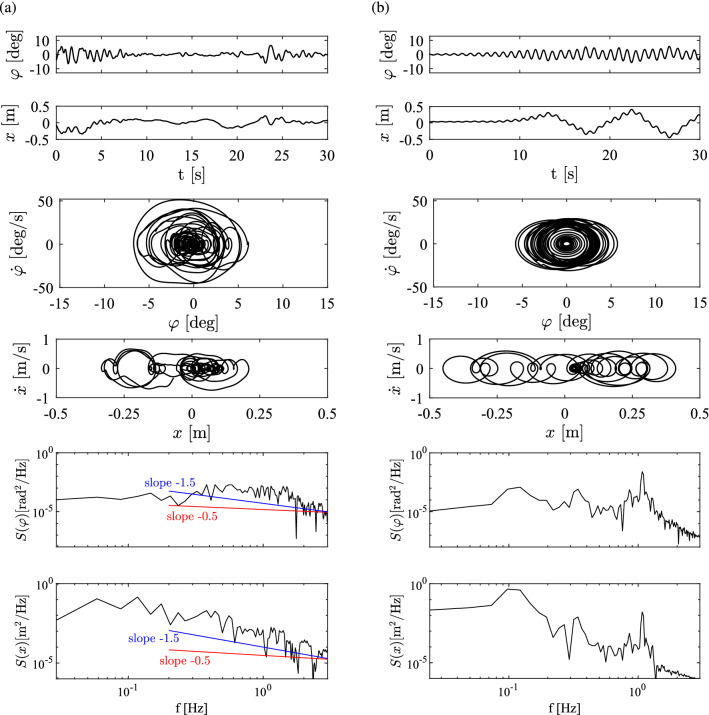
**a** Time histories, phase portraits and PSDs of $\varphi$ and *x* for a representative trial by S6 (MD subject). **b** Time histories, phase portraits and PSDs of the solution provided by the DSF model with parameters identified for S6. Parameters: $P_{\textrm{s}}=25.2, D_{\textrm{s}}=9.6, P_{\textrm{c}}=0.5, D_{\textrm{c}}=1, \varPi _{{\dot{\varphi }}}=0.1^{\circ }$/s, $\varPi _{\varphi }=0^{\circ }$, $\tau =170$ ms

### Participants

Thirty-one healthy individuals aged from 19 to 42 participated voluntarily in the study. Twenty-five subjects had no experience with this balancing task: they performed the balancing trials on the same day they first met the balancing device. These subjects are called *one-day (OD) subjects*. Six subjects exercised regularly for 15 weeks (at least 15 min/week) with the experimental setup before carrying out the measurements, which gave 5 h of accumulated balancing time for each subject. These subjects are referred to as *many-day (MD) subjects*. All subjects were free from neurological disease or upper limb injuries during the study, and did not take any medication which could affect motor control. Upon arrival to the laboratory, the subjects were informed about the experimental setup and protocol. The research was carried out following the principles of the Declaration of Helsinki and subjects were allowed to withdraw from the study at any time. The measurement lasted for an average of 15 min and subjects were allowed to rest if needed.

### Apparatus and procedure

The measurement setup is shown in Fig. 1a). The stick is pinned to the cart, which is allowed to move horizontally along a one-meter-long rail. Subjects were asked to sit in a chair so that their shoulders were parallel to the rail, and balance the stick using their dominant hand in the ML direction. All subjects were able to balance the 90 cm long stick for 30 s.

The motion of the stick was captured using an OptiTrack^®^ motion capturing system. The sampling frequency was set to 120 Hz. Two markers were attached to the stick, one close to the bottom and one at 40 cm distance from the bottom of the stick and, additionally, one marker was attached to the cart. Figure 3a shows the time histories of *x* and $\varphi$, the phase portraits in the plane $(\varphi , \dot{\varphi })$ and in $(x, \dot{x})$, and the power spectral densities (PSDs) for an OD subject. It has been suggested that the dynamics of human stick balancing exhibit intermittency (Cabrera and Milton 2002; Yoshikawa et al. 2016). Red and blue lines in the PSD diagrams show power laws with power law exponents of $-0.5$ and $-1.5$. For dynamical systems which exhibit intermittency, a power law exponent of $-0.5$ is observed in the power spectrum of the controlled variable (Cabrera et al. 2004) and an exponent of $-1.5$ is observed when the power law is determined for the laminar phases (Cabrera and Milton 2002; Yoshikawa et al. 2016). It can be seen that the PSDs in Fig. 3a do not exhibit these power law properties. Figure 3b shows the time signals, the phase portraits and the PSDs of the solution provided by the DSF model with parameters identified for this subject. The structure of both the measurement and simulation resemble to the cycles-within-cycles structure in Treffner and Kelso (1999).

Figure 4a shows the time histories *x* and $\varphi$, the corresponding phase portraits and PSDs for an MD subject. As can be seen, no power law can be observed, similarly to the OD subject. Figure 4b shows the time signals, the phase portraits and PSDs of the solution provided by the DSF model with parameters identified for this subject.

**Table 2:** Stabilometry parameters used to evaluate stick balancing measurements and simulations (Nagymáté et al. 2018)

Parameter	Unit	Description
$\sigma _{\varphi }$	rad	Standard deviation of the stick angle $\varphi$ or cart position *x*
$\sigma _x$	m	
MPF$_{\varphi }$	Hz	The mean power frequency is the weighted average frequency, calculated as $MPF=\frac{\sum _{j=1}^{M}f_jP_j}{\sum _{j=1}^{M}P_j}$, where $f_j$ are the frequency components, $P_j$ are their power and *M* is the number of frequency bits (Oskoei and Hu 2008)
MPF$_x$	Hz	
FD$_{\varphi }$	Hz	Frequency dispersion is the measure of variability of frequency content of power spectral density (Rocchi et al. 2004). Note that the FD quantity can be interpreted as the standard deviation of MPF, therefore it is determined as the 68% occupied bandwidth of the power spectral density (PSD) estimate
FD$_x$	Hz	
FP$_{\varphi }$	$\textrm{rad}^2/\textrm{Hz}$	Frequency power, which provides information about the power of the stick or cart movement in the frequency range 0.1–5 Hz
FP$_x$	$\textrm{mm}^2/\textrm{Hz}$	
FPR$_{\varphi }$	%	Frequency power ratio, which provides information about the power distribution of the stick or cart movement in the frequency range 0.1–1 Hz relative to the range 0.1–5 Hz
FPR$_x$	%	

### Data analysis

The high dimensionality and the sensitive dependence of the dynamics on the choice of initial condition make it difficult to estimate model parameters by comparing time series generated by the model to those observed experimentally. Here we assume that the model for pole balancing is acceptable if it reproduces the stabilometry properties shown in Table 2 that are determined for experimental pole balancing. Stabilometry was originally used as an objective tool to study body sway during quiet standing and during different balancing exercises (Petró et al. 2017; Nagymáté et al. 2018; Molnar and Insperger 2020; Molnar et al. 2021). The method is usually based on the analysis of the time variant center of pressure (CoP) coordinates (Kapteyn et al. 1983), however here we use stabilometry to investigate stick balancing. Table 2 lists the stabilometry parameters (SP), which were used in this study to evaluate human performance during stick balancing. Since the SPs are employed both for the stick angle $\varphi$ and the cart position *x*, altogether $2\times 5=10$ SPs are considered. One-way ANOVA was applied to determine differences in the SPs between MD and OD subjects.

Parameter identification was performed for all the 31 subjects for the DSF and PF feedback models. The SPs were used to compare the models and the measurements in order to identify the model parameters, which give time histories best matching the recorded balancing trials. For the parameter identification, a cost function *J* was defined, which compares the SPs for the simulated and measured time signals. The minimum of the cost function is determined in the 7 dimensional parameter space $(P_{\textrm{s}},\ D_{\textrm{s}},\ P_{\textrm{c}},\ D_{\textrm{c}},\ \varPi _{{\dot{\varphi }}}$, $\varPi _{\varphi }$, $\tau )$ with resolution shown in Table 1. The cost function is defined as

16 $$\begin{aligned} J=\sum _{i=1}^{N} w_{i} \left( \frac{\textrm{SP}_{i,\mathrm sim} - \textrm{SP}_{i,\mathrm meas}}{\textrm{SP}_{i,\mathrm meas}}\right) ^2, \end{aligned}$$

where *N* is the number of SPs selected for the parameter identification and $w_{i}$ denotes the weight of $\textrm{SP}_{i}$. Subscripts “sim” and “meas” refer to simulation and measurement, respectively. The six selected SPs were $\sigma _{\varphi }, \textrm{FP}_{\varphi }, \sigma _{x}, \textrm{MPF}_{x}, \textrm{FD}_{x}$ and $\textrm{FP}_{x}$ as will be discussed in Sect. 4.1, that is, $N=6$.

The weights applied in the cost function were determined based on the measurements as follows. Each SP was obtained for each of the 31 subjects individually, and the mean and standard deviation of each SP over the 31 subjects were evaluated. The coefficient of variation (Hansen et al. 1993) was obtained as the ratio of the standard deviation and the mean for each SP as

17 $$\begin{aligned} c_{\textrm{v},i}=\sigma (\textrm{SP}_{i,\mathrm meas})/\mu (\textrm{SP}_{i,\mathrm meas}), \quad i=1,2,\ldots , N. \end{aligned}$$

The weights in (16) can be given by

18 $$\begin{aligned} w_{i}=\frac{c_{\textrm{v},i}}{\sum _{i=1}^{N}c_{\textrm{v},i}}. \end{aligned}$$

This way, those SPs are taken into account with larger weights, which show higher variability (larger $c_{\textrm{v}}$) over the 31 subjects. The values of the weights are given in Table 3.

**Table 3:** Weights for stabilometry parameters applied in the cost function, estimated from the variability of stabilometry parameters over subjects

$w_{\sigma _{\varphi }}$	0.1502
$w_{\sigma _{x}}$	0.1003
$w_{\textrm{MPF}_{x}}$	0.0696
$w_{\textrm{FD}_{x}}$	0.1048
$w_{\textrm{FP}_{x}}$	0.2573
$w_{{FP_{\varphi }}}$	0.3179

## Results

Observations related to the human performance during stick balancing can be drawn based on the variation of SPs, which can be compared to those of the simulations obtained by the feedback models.

### Selection of stabilometry parameters

Once the subjects became familiar with the task, they usually could the stick within one or two trials without any difficulty. The $\sigma _{\varphi }$ and $\sigma _{x}$ parameters for the MD subjects are generally smaller than those of the OD stick balancers, which corresponds to the intuition that balancing performance improves with practice.

**Fig. 5:**
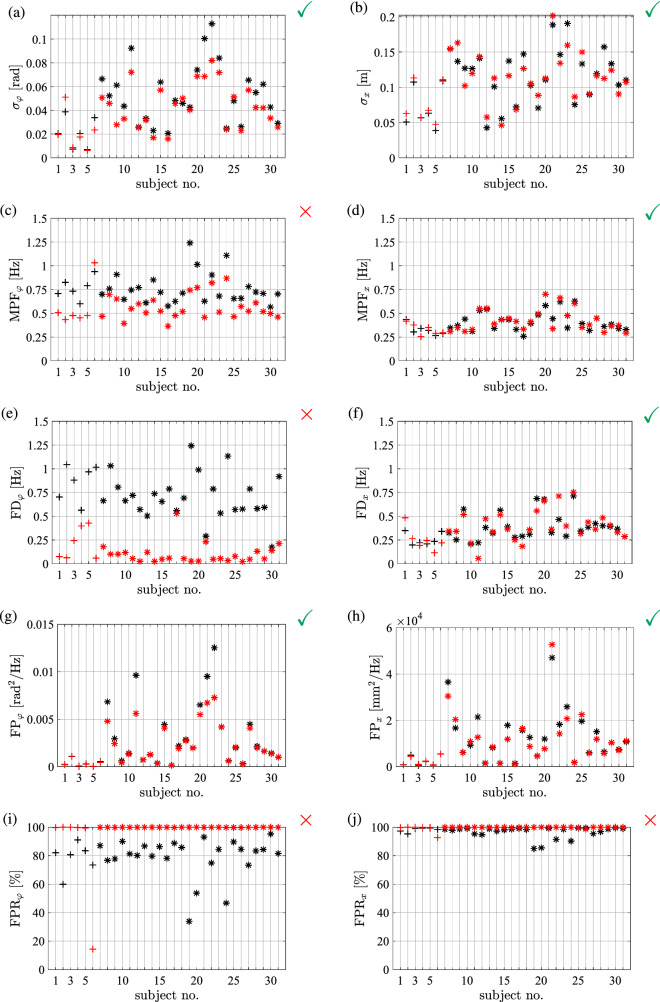
Stabilometry parameters determined from the measured time signals of $\varphi$ and *x* as a function of subject number. Symbol ‘+’ denotes values for many-day (MD) subjects, while ‘$*$’ denotes values for one-day (OD) subjects. Black markers show stabilometry values for measurements and red markers show stabilometry values obtained from simulations with the control parameters identified for each subject individually. Green check marks show significant difference ($p<0.05$) in the stabilometry parameters between MD (S1–S6) and OD balancers (S7–S31) while red cross depicts no significant difference

The SPs obtained from measurements (black) and from simulations with the identified parameters (red) are shown in Fig. 5a–j) as a function of subject number. The SPs related to the cart’s position *x* are directly affected by the control action of the human subjects, while the SPs for the stick’s position $\varphi$ involve the dynamics of the inverted pendulum, too. The variability in the SPs for $\varphi$ is higher than those for *x*.

One-way ANOVA was applied to determine differences between MD and OD balancers with respect to SPs. ANOVA revealed significant differences ($p<0.05$) for $\sigma _{\varphi }, \textrm{FP}_{\varphi }, \sigma _{x}$, $\textrm{MPF}_{x}, \textrm{FD}_{x}$ and $\textrm{FP}_{x}$, as shown in Fig. 5 with green check marks. These parameters are considered as reliable indicators to distinguish between balancing performance levels and therefore these are applied in the cost function *J* in Eq. (16) to compare simulations and measurements.

### Parameter identification

The minimum of the cost function over the 7 dimensional parameter space $(P_{\textrm{s}},\ D_{\textrm{s}},\ P_{\textrm{c}},\ D_{\textrm{c}},\ \varPi _{{\dot{\varphi }}}$, $\varPi _{\varphi }$, $\tau )$ is searched numerically with resolution given in Table 1 for each subject. The cost function value was determined for both DSF and PF control models and for each subject.

**Fig. 6:**
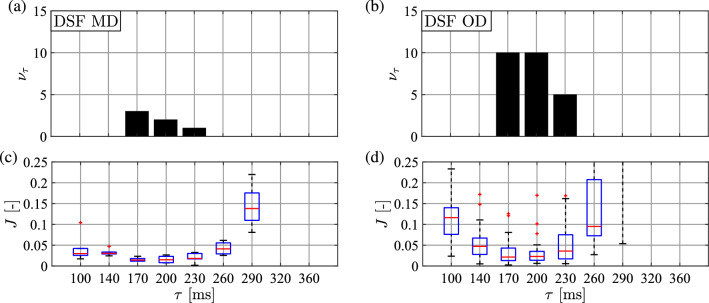
Top: distribution of the identified $\tau$ values based on the minimum of the cost function for **a** many-day (MD) and for **b** one-day (OD) subjects for the DSF model. Bottom: boxplot for the cost function values as a function of the applied time delays for **c** MD and for **d** OD subjects. Red central mark: median; blue box: interquartile range (IQR); black dashed whiskers: min–max values not considered outliers; red $+$ marks: outliers

**Fig. 7:**
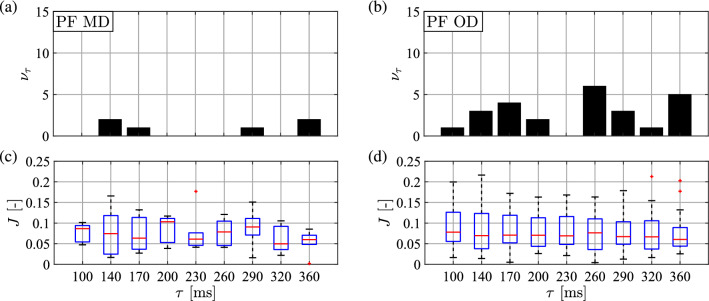
Top: distribution of the identified $\tau$ values based on the minimum of the cost function for **a** many-day (MD) and for **b** one-day (OD) subjects for the PF model. Bottom: boxplot for the cost function values as a function of the applied time delays for **c** MD and for **d** OD subjects. Boxplot notation is the same as in Fig. 6

Figures 6 and 7 show the result of the parameter identification in terms of the delay $\tau$ for the DSF and the PF models, respectively. Panels (a) and (b) in both figures present the distribution of the delays identified for the MD and OD subjects. For all subjects the identified delays were between 170 and 230 ms for the DSF model. For the PF model, the distribution of the identified delay is irregular and sparse, which may be caused by the invalidity of the PF model.

The variation of the cost function *J* over the time delays for MD and OD subjects is shown in panels (c) and (d) of Figs. 6 and 7 for DSF and PF models, respectively. For the DSF model, it can be seen that the median of the cost function is minimal between $\tau =170-230$ ms. The cost function median gets larger for other time delays, especially for delays larger than $\tau =260$ ms. For the PF model, there is no significant difference in the variation of cost function as a function of the reaction delay and the cost function values are high for all time delays. The above observations support the validity of the DSF model against the PF one.

The average of the identified parameters $P_{\textrm{s}}$, $D_{\textrm{s}}$, $P_{\textrm{c}}$, $D_{\textrm{c}}$, $\varPi _{{\dot{\varphi }}}$, $\ \varPi _{\varphi }$ and $\tau$ for the DSF model where the cost function *J* is minimal are shown in Table 4 separately for MD and OD subjects. The time delay for both groups is about 200 ms, which is slightly smaller than those measured for balancing on the fingertip (Mehta and Schaal 2002; Milton et al. 2016). This may be due to the additional processing time required for the depth perception in the AP direction during fingertip stick balancing.

**Table 4:** Mean and standard deviation of the identified control parameters and the minima of the cost functions for many-day (S1–S6) and one-day (S7–S31) subjects for the DSF model

	$P_{\textrm{s}}$	$D_{\textrm{s}}$	$P_{\textrm{c}}$	$D_{\textrm{c}}$	$\varPi _{{{\dot{\varphi }}}}$	$\varPi _{{\varphi }}$	$\tau$	$J_{\textrm{min}}$
Subject group	[N/rad]	[Ns/rad]	[N/m]	[Ns/m]	[$^{\circ }$/s]	[$^{\circ }$]	[ms]	[−]
MD mean	23.07	6.93	0.225$$^*$$	0.758	0.053$$^{**}$$	0.052	190	0.012
MD std	1.938	1.78	0.214	0.400	0.060	0.043	24.50	0.007
OD mean	23.86	6.356	0.480$$^*$$	0.668	0.251$$^{**}$$	0.041	194	0.023
OD std	2.360	0.936	0.291	0.330	0.223	0.042	22.91	0.024

Symbols $$^*$$ and $$^{**}$$ show significant difference in the identified parameters between MD and OD subjects

ANOVA detects no significant differences between parameters identified for MD and OD subjects in terms of $P_{\textrm{s}},$ $D_{\textrm{s}},\ D_{\textrm{c}},\ \varPi _{\varphi }$, $\tau$ and the cost function *J*. However, there is significant difference for $P_{\textrm{c}}$ and $\varPi _{{\dot{\varphi }}}$ ($p<0.05$), both being generally smaller for MD subjects.

**Table 5:** Validation of the parameter identification method. The identified DSF mean parameters match well the simulation parameters

	$P_{\textrm{s}}$, ${\hat{P}}_{\textrm{s}}$	$D_{\textrm{s}}$, ${\hat{D}}_{\textrm{s}}$	$P_{\textrm{c}}$, ${\hat{P}}_{\textrm{c}}$	$D_{\textrm{c}}$, ${\hat{D}}_{\textrm{c}}$	$\varPi _{{{\dot{\varphi }}}}$	$\varPi _{{\varphi }}$	$\tau$
	[N/rad]	[Ns/rad]	[N/m]	[Ns/m]	[$^{\circ }$/s]	[$^{\circ }$]	[ms]
Simulation parameters	23.86	6.356	0.480	0.668	0.251	0.041	194
PF mean	32.74	2.968	0.911	0.790	0.117	0.082	304
PF std	14.37	1.779	0.276	0.373	0.156	0.038	58.41
DSF mean	23.05	7.123	0.419	0.871	0.232	0.066	187
DSF std	1.877	0.989	0.211	0.222	0.208	0.037	24.11

**Fig. 8:**
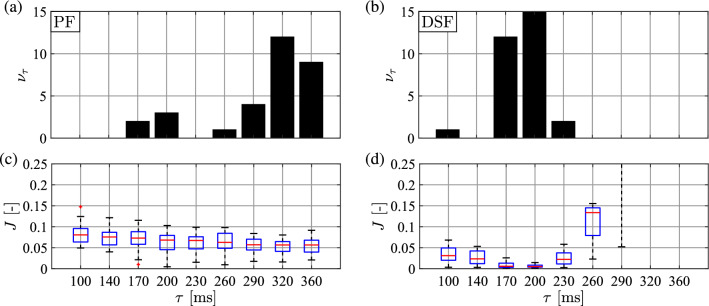
Validation of the parameter identification method using simulation with the identified OD parameters of the DSF model. Top: distribution of the identified $\tau$ values based on the minimum of the cost function for **a** PF and **b** DSF model. Bottom: boxplot for the cost function values as a function of the applied time delays for **c** PF and for **d** DSF model. Boxplot notation is the same as in Fig. 6

### Validation of the parameter identification

In order to validate the results, the same parameter identification method was performed for the DSF and PF models using time-domain simulations with the mean OD parameters in Table 4 with 31 different initial conditions. The results are shown in Table 5 and Fig. 8. The simulation parameters have been accurately identified for the DSF model with identified delay 187 ms (see Table 5). For the PF model, the distribution of the identified delays is irregular and the cost function shows no clear minimum (see Fig. 8) as expected. This validates the parameter identification method.

As an additional validation, the stabilometry parameters were determined for time domain simulations generated by employing the identified parameters for all subjects. The resulted SPs are shown in Fig. 5 by red markers. As can be seen, for the selected SPs ($\sigma _{\varphi }, \textrm{FP}_{\varphi }, \sigma _{x}$, $\textrm{MPF}_{x}, \textrm{FD}_{x}$ and $\textrm{FP}_{x}$ all indicated by green check mark in Fig. 5) obtained from the measurements and from the simulations show similar distribution. This means that the DSF control model generates quantitatively very similar results to the measurements. Especially remarkable agreement has been observed between the measurements and the simulations for the MD subjects in terms of the parameters $\text {FP}_{\varphi }$ and $\text {FP}_{x}$ . The red and black ‘+’ symbols practically coincide in panels (g) and (h).

### Difference between OD and MD subjects

**Fig. 9:**
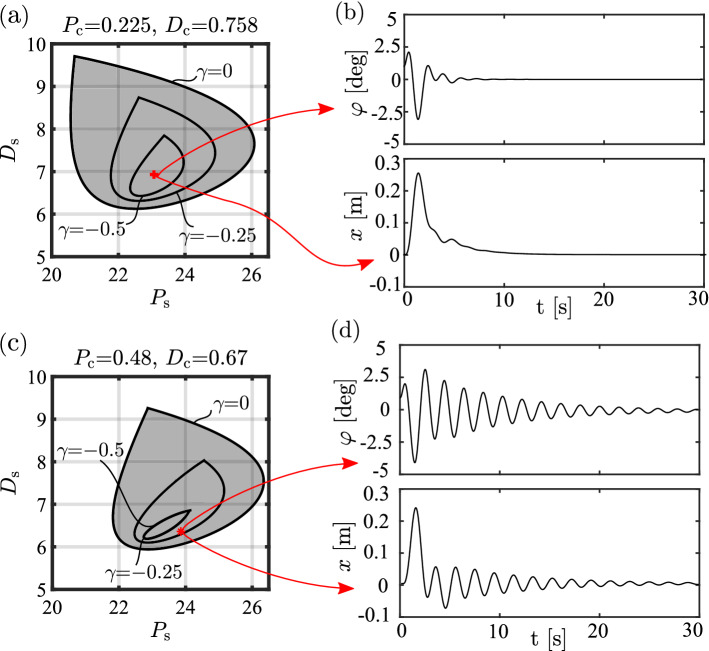
Left panels: linear stability diagrams of **a** MD and **c** OD subjects, symbol ‘+’ denotes the estimated $P_{\textrm{s}}$ and $D_{\textrm{s}}$ values for MD, while ‘$*$’ denotes the estimated $P_{\textrm{s}}$ and $D_{\textrm{s}}$ values for OD subjects. Grey shading indicates stable regions and contour curves indicate different exponential decay rates $\gamma$. Right panels: the corresponding time histories for **b** MD and **d** OD subjects (without dead zone applied in the model)

The difference between the control gains of OD and MD subjects can be visualized in stability diagrams shown in Fig. 9. Note that these diagrams reflect the dynamics without a dead zone, hence in the stable region the solution decays exponentially such that

19 $$\begin{aligned} |\varphi (t)| \lessapprox \varphi (0) e^{\gamma t}, \end{aligned}$$

where $\gamma < 0$ is the exponential decay rate (Michiels and Niculescu 2007; Insperger and Milton 2021). Therefore, $\gamma$ represents the settling time: the smaller $\gamma$ the faster the decay. The average of the identified parameters are shown both for MD and OD subjects in Fig. 9a and c with markers ‘+’ and ‘$*$’, respectively. It can be seen that for MD subjects, the average control gains are located in the inside of the stable region corresponding to smaller $\gamma$ values, hence faster settling. For OD subjects, the average control gains are located close to the edge of the stability regions. The corresponding time histories are shown in Fig. 9 for (b) MD and (d) OD subjects (without thresholds). It can be seen that the solution decays faster for MD subjects ($\gamma _{\textrm{MD}}=-0.75~\hbox {s}^{-1}$) than for ODs ($\gamma _{\textrm{OD}}=-0.3~\hbox {s}^{-1}>\gamma _{\textrm{MD}}$). This might explain the difference between $\sigma _{\varphi }$ and $\sigma _{x}$ for the MD and OD subjects.

## Conclusions

Stick balancing on a linear track was investigated via stabilometry parameters, where subjects with many-day (MD) and one-day (OD) experience were asked to carry out the balancing task. Standard deviations of stick angle and cart positions were smaller for MD than for OD subjects. The frequency power was also smaller for MDs which is related to the fact that MD subjects can perform the balancing on a lower energy level, i.e., more effortlessly (Milton et al. 2007).

Two control force models (DSF and PF) were investigated via numerical brute force method, and stabilometry parameters of the resulting time histories were analyzed. Both control force models apply a proportional-derivative (PD) controller with respect to the stick and the cart, which is switched on and off based on threshold crossing conditions. The domain of stable control gains for the stick’s position and velocity shrinks with increasing gains for the cart position and velocity.

The control parameters $P_{\textrm{s}},\ D_{\textrm{s}},\ P_{\textrm{c}},\ D_{\textrm{c}}$, the thresholds $\varPi _{\varphi }$, $\varPi _{{\dot{\varphi }}}$, and the reaction delay $\tau$ were identified for all the 31 subjects based on a cost function, which was defined as the weighted sum of the relative error between stabilometry parameters of measured and simulated data. Parameter estimation showed that the DSF model fits better to the measurements for both MDs and ODs. In terms of the internal model representation, this means that the contribution of the integral term in (6) is very small. Note that balancing shorter sticks is more challenging and expert stick balancers may develop a PF strategy for these shorter sticks (Milton et al. 2016).

For the DSF model it was found that MD subjects have significantly smaller $P_{\textrm{c}}$ and $\varPi _{{\dot{\varphi }}}$ values than ODs. It is plausible that the nervous system finds it easier to estimate threshold for angle and would take some experience before it could make a good estimate of threshold for angular velocity (i.e., thresholds for angular velocity can be lowered by experience Werkhoven and Koenderink 1991; Barazza and Grzywacz 2003; Nijhawan and Wu 2009). As a result, MD subjects use less effort to control the position of the cart, which is a secondary control task after controlling the angle of the stick, therefore they are able to apply smaller $P_{\textrm{c}}$ gains.

A surprising observation is that balancing a stick on a linear track reduces $\varPi _{\varphi }$ by 20–60 times the value observed for stick balancing at the fingertip, but does not completely eliminate it. Moreover, $\varPi _{\varphi }$ is 2–3 times larger than the resolution of the visual system ($0.02^{\circ }$ DeValois and DeValois 1990). From a control theoretic perspective, an advantage of a detection threshold is that it reduces the destabilizing effects of overcontrol, which occurs in noisy time-delayed dynamical systems (Milton et al. 2008). A consequence is that the detection threshold becomes a control parameter. Thus it might be better to consider the detection threshold to a *certainty threshold*, i.e., when the sensory variable is above this threshold, the nervous system must certainly make a control action.

Track balancing in the ML direction can be linked to virtual balancing tasks, where subjects control the movement of an object represented on a computer screen inherently in the ML direction (Loram et al. 2011; Zgonnikov et al. 2014; Bazzi et al. 2018). In these virtual tasks, it is also possible to artificially modify the conditions of the balancing task, e.g., extra delay can be added in the feedback loop (Kovacs et al. 2019; Franklin et al. 2019) or other than Newtonian dynamics can be implemented (Kovacs and Insperger 2022).

It shall be mentioned that several other types of control concepts exist in the literature to capture the dynamics of human stick balancing. The intermittent control developed for human quiet standing in Asai et al. (2009) was adopted to human stick balancing in Yoshikawa et al. (2016). The main idea of the intermittent control is that the feedback is switched off if the state is close to the stable manifold of the uncontrolled system. Other time-dependent control concepts, such as the clock-driven intermittent control (Gawthrop et al. 2013) or the act-and-wait control (Insperger and Milton 2014) has also been showed up as possible mechanisms for human stick balancing. Extending the feedback by an acceleration term was shown to reduce the destabilizing effect of the feedback delay (Insperger and Milton 2014). The common feature of the above controllers is that they all are based on DSF, i.e., they employ a feedback of the position and the velocity in a sophisticated manner. Similarly to intermittent controller, DSF with well-tuned control gains can exploit the stable manifold of the uncontrolled system as the proportional and the derivative terms cancel each other if the state is close the stable manifold. In this paper therefore we rather concentrated on two main concepts, whether the control directly uses the most recent available information for error correction (DSF), or it attempts to predict the effect of previous control actions (PF).

Taken together, our results suggest that when important sensory information is missing the only recourse for controlling balance is to predict the sensory consequences of the movement. However, this is not the case in track balancing, where the sensory dead zones for the stick’s position are much smaller than for stick balancing at the fingertip. Our studies show that these dead zones are small enough so that track balancing of a long enough stick ($\sim 90$ cm) can be well modeled with delayed state feedback. This demonstration points to the importance of determining the availability and reliability of sensory information for understanding how motor control is exerted by the nervous system.
